# Effects of Transporter Inhibitors and Chemical Analogs on the Uptake of Antimonite and Antimonate by *Boehmeria nivea* L.

**DOI:** 10.3390/toxics11100860

**Published:** 2023-10-14

**Authors:** Yi Lu, Fangyuan Peng, Yingyang Wang, Haipu Li, Zhaoguang Yang

**Affiliations:** 1 Center for Environment and Water Resource, College of Chemistry and Chemical Engineering, Central South University, Changsha 410083, China; 2 Key Laboratory of Hunan Province for Water Environment and Agriculture Product Safety, Changsha 410083, China

**Keywords:** ramie, antimony, uptake pathway, aquaglyceroporin

## Abstract

Antimony (Sb) is a non-essential metalloid that can be taken up by plants from contaminated soils and thus enter the food chain and threaten human health. *Boehmeria nivea* L. (ramie) is a promising phytoremediation plant for Sb-polluted soils. However, the mechanisms of antimonite (SbIII) and antimonate (SbV) uptake by ramie remain unclear. In this study, a hydroponic system was established to investigate how different substances affect the uptake of SbIII or SbV by ramie, including an energy inhibitor (malonic acid), an aquaglyceroporin inhibitor (silver nitrate), an SbV analog (phosphate—PV), and SbIII analogs (arsenite—AsIII, glycerol, silicic acid—Si, and glucose). The results indicated that ramie primarily transported Sb by increasing the Sb concentration in the bleeding sap, rather than increasing the weight of the bleeding sap. After 16 h of Sb exposure, the absolute amount of transported Sb from the roots to the aboveground parts was 1.90 times higher under SbIII than under SbV. The addition of malonic acid significantly inhibited the uptake of SbV but had limited effects on SbIII, indicating that SbV uptake was energy dependent. PV addition significantly reduced SbV uptake, while the addition of AsIII, glycerol, and Si obviously inhibited SbIII uptake. This suggested that the uptake of SbV might be via low-affinity P transporters and SbIII might use aquaglyceroporins. These findings deepen the understanding of Sb uptake pathways in ramie, contribute to a better comprehension of Sb toxicity mechanisms in ramie, and establish a foundation for identifying the most effective Sb uptake pathways, which could further improve the efficiency of phytoremediation of Sb-polluted soils.

## 1. Introduction

Antimony (Sb), a non-essential metalloid with no recognized biological role [1], is recognized as a priority environmental contaminant by both the United States Environmental Protection Agency and the European Union [2,3]. Sb belongs to the same group in the periodic table as phosphorus (P) and arsenic (As). Similar to As, Sb exists in oxidation states of +3 and +5 in the environment, with antimonite (SbIII) dominating under anoxic conditions and antimonate (SbV) prevailing in oxic environments [4]. Despite being non-essential, plants can take up Sb from contaminated soil, which can lead to potential human exposure through the consumption of Sb-contaminated crops, thereby posing health risks, including an increased risk of cancer and respiratory disorders [1,5,6]. Soil samples from contaminated areas have exhibited remarkably high Sb concentrations, often exceeding several thousand mg/kg in proximity to mining sites [1,7,8,9]. These levels far exceed the soil Sb concentration limit of 36 mg/kg established by the World Health Organization [10]. Phytoremediation, as an effective biotechnology, maintains the soil structure at the remediation site, minimizes secondary contamination of groundwater, and prevents secondary transport of heavy metals through wind and water erosion [11,12]. Our previous studies have established that *Boehmeria nivea* L. (ramie) exhibits remarkable tolerance to Sb and possesses a significant capacity for Sb accumulation [13,14]. This makes ramie an excellent candidate for phytoremediation purposes in Sb-contaminated soil. Studying the mechanisms of Sb uptake by ramie can help identify the most effective uptake channels, thereby improving the effectiveness of phytoremediation. However, the mechanisms underlying the uptake of Sb by plants remain inadequately understood.

Currently, there is limited information on whether there is an energy cost for the uptake of SbIII or SbV in plants. The tricarboxylic acid (TCA) cycle plays a vital role in metabolism, serving as a crucial pathway for generating adenosine triphosphate (ATP) and providing precursors for various biosynthetic processes [15,16]. Succinate dehydrogenase (SDH), a vital component of the TCA cycle [17], can be effectively inhibited by malonic acid (C_3_H_4_O_4_) [18]. Consequently, the presence of malonic acid disrupts the TCA cycle, resulting in a reduction in the production of ATP. Feng et al. [19] employed C_3_H_4_O_4_ as an inhibitor and found that the uptake of SbV in rice might be energy dependent. However, whether the process of Sb uptake by ramie involves energy expenditure remains to be elucidated. 

The mechanisms for the uptake of SbV in plants are not yet fully understood. It has been suggested that SbV, similar to AsV, may be taken up by plants through phosphate (PV) transporters [20]. In support of this, Feng et al. [19] found a significant reduction in SbV uptake in rice when PV was added, indicating a competitive interaction between PV and SbV for the same uptake pathway. However, contradictory evidence suggests that plants taking up SbV may not utilize the PV pathway. For instance, Tschan et al. [21] reported that the addition of PV did not inhibit SbV uptake in sunflower plants (*Helianthus annuus*) or maize (*Zea mays*), leading to the inference that plants might not use the PV uptake system for SbV uptake. This disparity could be attributed to the structural differences between PV and SbV, with PV having a tetrahedral structure while SbV possesses an octahedral structure, resulting in a larger molecular size and lower charge density [1,21,22,23]. In order to gain a deeper understanding of the mechanism of SbV uptake in ramie, further studies must be conducted to elucidate whether SbV competes with PV for uptake pathways in ramie.

Aquaporins can be categorized into two main groups: water-specific channels, and aquaglyceroporins that facilitate the transport of glycerol, water, and other small, uncharged solutes [24]. Aquaglyceroporin channels serve as transporters for glycerol and metalloids, such as AsIII, SbIII, silicic acid, and boric acid, which share structural similarities with glycerol [24]. Meharg et al. [25] found dose-dependent competition between AsIII, SbIII, and glycerol for uptake in paddy rice (*Oryza sativa*), suggesting a shared uptake mechanism through aquaglyceroporin transport channels. Plant aquaporins belonging to the nodulin 26-like intrinsic protein (NIP) subfamily have the capability to transport SbIII. Bhattacharjee et al. [24] found that Arabidopsis thaliana employed aquaglyceroporin channels for the uptake of SbIII and AsIII, with distinct preferences: AtNIP5;1 and AtNIP6;1 favored AsIII, while AtNIP7;1 preferred SbIII. Kamiya et al. [26] also identified NIP1;1 as an SbIII transporter in Arabidopsis thaliana. Ag is an effective aquaglyceroporin inhibitor by interacting with a cysteine near the conserved NPA motif, obstructing the constriction region [27]. However, Tisarum et al. [28] found that aquaglyceroporin competitors like glycerol, glucose, silicic acid, and AsIII did not inhibit SbIII uptake, and Ag had no effect on its transport in *Pteris vittata* gametophytes. Thus, aquaglyceroporins from various plants appear to be selective for SbIII uptake. Whether SbIII uptake in ramie is via aquaglyceroporins needs to be verified.

This study aimed to reveal the uptake mechanisms of SbIII and SbV by ramie. The specific objectives were to investigate (1) the differences in the translocation of SbIII and SbV from the roots to the shoots by monitoring bleeding from sap xylem; (2) the effects of an energy inhibitor and aquaglyceroporin inhibitor on the uptake of SbIII and SbV; (3) the effects of SbV analogs of PV on SbV uptake; and (4) the effects of SbIII analogs including AsIII, glycerol, silicic acid, and glucose on SbIII uptake. The findings are expected to advance our understanding of the mechanisms of Sb uptake in ramie, thus shedding light on the complexity of Sb toxicity in ramie. Furthermore, these insights can help to identify the most effective uptake pathways and ultimately improve phytoremediation strategies for Sb-contaminated soils.

## 2. Materials and Methods

### 2.1. Plant Materials and Treatments

Ramie seedlings (Zhongzhu No.1) were obtained from a cultivation center in Hunan, China. Ramie shoots, measuring approximately 15 cm in length and complete with leaves, were chosen and planted in 20% Hoagland solutions to promote root development. These plants were cultivated in a greenhouse with a light cycle of 16 h (around 28 °C) and a dark cycle of 8 h (approximately 20 °C), maintaining a relative humidity level of 60–70%. The nutrient solution was refreshed on a weekly basis. After 21 days of growth, reaching a height of approximately 25 cm, the plants with similar growth conditions were selected, washed with ultrapure water, and transplanted into plastic pots filled with 2 L of 50% Hoagland solution.

Energy inhibitors (malonic acid, C_3_H_4_O_4_, Sigma-Aldrich, St. Louis, MO, USA) were mixed at concentrations of 1, 5, and 10 mg/L, along with either 10 mg/L SbIII or 10 mg/L SbV. In the case of silver (Ag) treatments, silver nitrate (AgNO_3_, Sigma-Aldrich, USA) was introduced at concentrations of 0.01, 0.1, and 1 mg/L to the culture solutions, followed by the application of 10 mg/L SbIII or SbV after 1 h to allow for prior aquaglyceroporin inhibition. As a competitor for SbV, PV (KH_2_PO_4_, Aladdin Reagent Company, Shanghai, China) was added at concentrations of 1 and 10 mg/L to the culture solution containing 10 mg/L SbV. For SbIII competitors, AsIII (NaAsO_2_, Sigma-Aldrich, USA), glycerol (C_3_H_5_(OH)_3_, Aladdin Reagent Company, Shanghai, China), silicic acid (Na_2_SiO_3_·5H_2_O, Aladdin Reagent Company, Shanghai, China), and glucose (CH_2_OH(CHOH)_4_HCO, Aladdin Reagent Company, Shanghai, China) were introduced at concentrations of 1 and 10 mg/L to the culture solution containing 10 mg/L SbIII. To prevent silicic acid polymerization, 3 mM of 2-(Nmorpholino) ethanesulfonic acid (MES, Aladdin Reagent Company, Shanghai, China) was added to the Si treatments [29]. SbIII and SbV were added in the form of potassium antimony tartrate (C_8_H_4_K_2_O_12_Sb_2_·3H_2_O, Aladdin Reagent Company, Shanghai, China) and potassium hexahydroxoantimonate (KSb(OH)_6_, Sigma-Aldrich), respectively. The pH of all treatment solutions was adjusted to 6.3. There were a total of 25 treatment groups, as indicated in Table 1. Each treatment group consisted of three replicate pots, with each plastic pot containing three ramie plants. The three ramie plants in each plastic pot were mixed to create a composite sample. The growth conditions were maintained in accordance with the previous settings.

After 3 days of exposure, the ramie plants were harvested. Ramie roots were rinsed and immersed in a solution of 20 mM Na_2_-EDTA for 30 min to eliminate surface-bound Sb [30]. Subsequently, the plants were thoroughly rinsed with ultrapure water, and the surface water was absorbed by filter paper. They were then carefully divided into roots and leaves. Afterward, the plant samples were freeze-dried and ground into fine powders.

### 2.2. Collection of Bleeding Sap

The collection of bleeding sap followed the process outlined by Feng et al. [19]. The culture process of ramie was the same as that described above. There were a total of three treatment groups, comprising the control, SbIII (10 mg/L), and SbV (10 mg/L). Each treatment was replicated three times. After introducing SbIII and SbV for a duration of one day, the upper portions of three seedlings were cut, leaving approximately 2 to 3 cm above the root section. The cut surface was rinsed with ultrapure water and dried using filter paper. Subsequently, the cross-section was encircled with weighed degreasing cotton (wrapped in plastic wrap). After sixteen hours, the degreasing cotton was re-weighed and then subjected to digestion for Sb concentration determination. The weight of xylem sap bleeding was calculated as the difference between the two weight measurements. The absolute amount of Sb transported from the roots to the aboveground parts was calculated as follows [31]:

Sb transported (ng/h) = Sap flow (g/h) × Sb concentration in sap (mg/kg)(1)

### 2.3. Sample Digestion

The sample digestion process followed the microwave-assisted acid digestion meth-od [32]. Briefly, either degreased cotton or 0.2 g of finely ground plant sample was accurately weighed and then placed into a digestion vessel, to which, 8 mL of HNO_3_ and 2 mL of H_2_O_2_ were added. Subsequently, a microwave digestion system (MDS-6G; Sineo Microwave Chemistry Technology Co., Ltd., Shanghai, China) was applied to digest the mixtures, maintaining a temperature of 190 °C for a duration of 30 min. Once the digestion process was complete, the solution was cooled to room temperature, and it was then diluted to 50 mL using ultrapure water. The resulting solution was then filtered through 0.45 μm cellulose acetate membranes and stored at 4 °C for subsequent analysis.

### 2.4. Determination of Sb and Ag Concentrations

Inductively coupled plasma optical emission spectrometry (ICP-OES, Optima 8000, PerkinElmer, Waltham, MA, USA) was utilized to determine Sb concentrations following the USEPA SW-846 Test Method 6010D (USEPA, 2018). For the detection of Ag concentrations, inductively coupled plasma mass spectrometry (ICP-MS) (7700×; Agilent Corp., Santa Clara, CA, USA) was used in accordance with USEPA Method 6020B (USEPA, 2014).

### 2.5. Statistical Analysis

The data are expressed as the mean ± standard deviation (SD) (*n* = 3). To evaluate the variations among treatments, one-way analysis of variance (ANOVA) was conducted, followed by either the least significant difference (LSD) test or the Games–Howell test, depending on the homogeneity of variances, at a significance level of *p* < 0.05. The statistical analysis was performed by SPSS 26 (IBM, New York, USA) for Windows, and data visualization was carried out by Origin 2021(OriginLab, Northampton, MA, USA).

## 3. Results and Discussion

### 3.1. Xylem Transport of Sb

Table 2 shows that, compared to the control group, 10 mg/L SbIII or SbV exposure did not significantly affect the weight of the bleeding sap. The weight of bleeding sap is closely related to root pressure and is typically considered to be an indicator of root activity [33]. The similar bleeding weight among the treatment groups in this study indicated that 10 mg/L SbIII or SbV had a limited effect on the root activity of ramie. In contrast, Feng et al. [19] reported a slight discrepancy in their results. They found that, in rice, SbIII did not significantly affect the weight of bleeding sap comparing with the control group, while SbV exposure led to a significant increase in sap weight. This variation could be attributed to their use of higher exposure concentrations and potential differences in the transport capabilities of different plant species for various Sb species. However, it is noteworthy that exposure to both Sb species significantly increased the Sb concentrations in the bleeding sap, with the SbIII treatment group exhibiting more than double the concentration of the SbV treatment group. This might be due to the fact that the neutral SbIII (Sb(OH)_3_) crossed the negatively charged root surface into the plant more easily than the negatively charged SbV anion (Sb(OH)_6_^–^) [34]. Previous studies demonstrated that multiple physiological processes contribute to the enhanced accumulation of Cd in the shoots, with efficient root uptake and proficient xylem transport being prominent factors [32,35,36]. Uroic et al. [31] demonstrated that the weight of bleeding sap and the concentration of As in the xylem sap actively determine the actual transport amount of As in the xylem. Based on Formula (1), after 16 h of Sb exposure, the absolute amount of Sb transported from the roots to the aboveground parts was 1.90 times greater under SbIII stress compared to the amount transported under SbV exposure. This finding aligns with our earlier results [14], where the Sb levels in the SbIII-treated ramie leaves were remarkably higher than those observed under SbV exposure. This suggests that ramie primarily transports Sb from its roots to the aboveground parts by increasing the concentration of Sb in the bleeding sap, and ramie demonstrated greater effectiveness in the uptake of SbIII compared to SbV.

### 3.2. Effects of Malonic Acid on Sb Uptake

**Figure 1:**
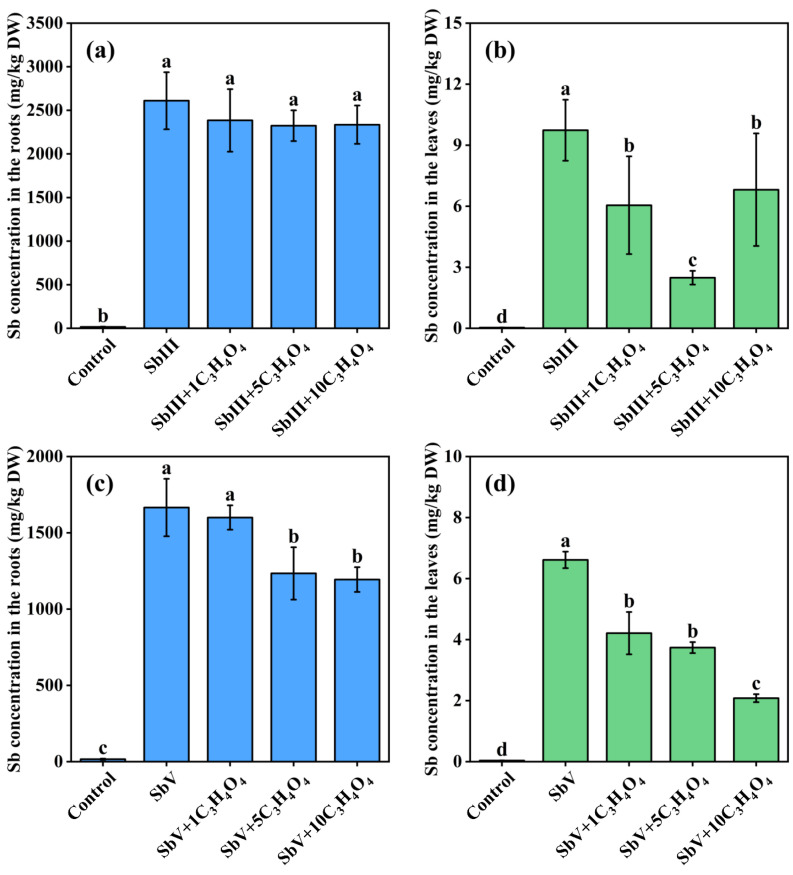
Effects of C_3_H_4_O_4_ on Sb concentrations in the SbIII-treated roots (**a**), SbIII-treated leaves (**b**), SbV-treated roots (**c**), and SbV-treated leaves (**d**). Lowercase letters refer to statistically significant differences at *p* < 0.05. DW: Dry weight.

Figure 1 illustrates the effects of malonic acid on Sb uptake by ramie. When ramie was exposed to SbIII, the Sb levels in the roots remained relatively stable with the addition of C_3_H_4_O_4_, while the Sb content in the leaves showed a significant reduction. In contrast, the addition of C_3_H_4_O_4_ had a marked effect on decreasing the Sb concentrations in both the roots and leaves under SbV stress. Furthermore, the reduction in Sb levels became more pronounced as the concentration of C_3_H_4_O_4_ increased. Similarly, Feng et al. [19] observed that the addition of C_3_H_4_O_4_ inhibited the uptake of SbV but had little effect on SbIII in rice, suggesting that the uptake of SbV by rice may require energy consumption. Arsenic, as an analog of Sb in the same group, shares some similarities in its behavior. Lei et al. [37] demonstrated that the translocation of arsenate (AsV) in the As hyperaccumulator Pteris vittata was an active process that required energy. Therefore, the suppression of SbV uptake due to the addition of C_3_H_4_O_4_ suggested that the uptake of SbV by ramie was energy dependent and required ATP for energy supply. Furthermore, the suppressed Sb concentrations in ramie leaves under SbIII stress by C_3_H_4_O_4_ could be attributed to our prior research findings indicating that SbIII was mostly oxidized to SbV in ramie leaves after uptake [12]. Whether the uptake and transport of SbIII require energy requires further investigation.

### 3.3. Aquaglyceroporin Inhibitor on Sb Uptake+

**Figure 2:**
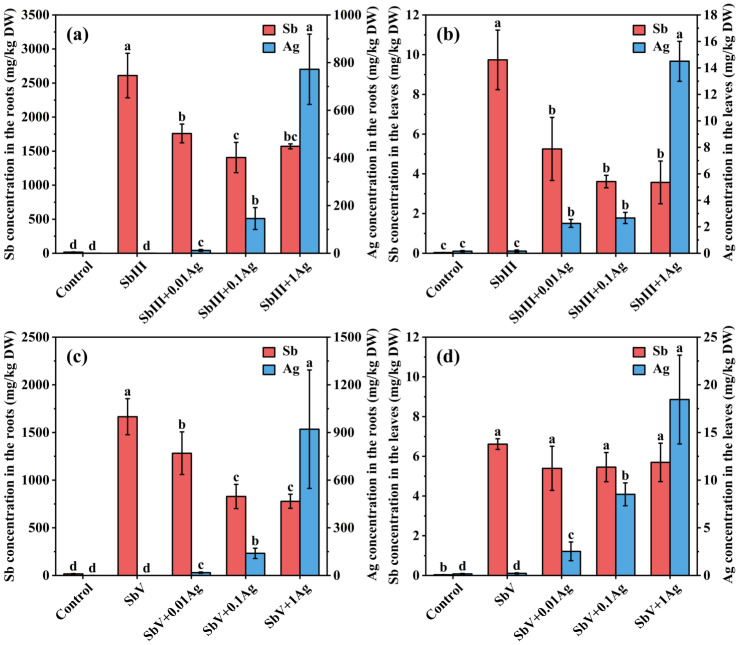
Effects of Ag on Sb concentrations in the SbIII-treated roots (**a**), SbIII-treated leaves (**b**), SbV-treated roots (**c**), and SbV-treated leaves (**d**). Lowercase letters indicate statistically significant differences at *p* < 0.05. DW: Dry weight.

The effects of silver (Ag) on Sb levels in ramie tissues under SbIII and SbV stresses were investigated to explore whether ramie plants take up Sb through aquaglyceroporin. Ag is commonly employed as an aquaglyceroporin inhibitor due to its interaction with the sulfhydryl group of a cysteine situated in the proximity of the conserved NPA motif, effectively obstructing the constriction region of the aquaglyceroporin [27]. Compared to the widely used mercury (Hg)-containing compounds for aquaglyceroporin inhibition, Ag inhibition is more potent and specific and poses lower toxicity concerns [27,28]. This is particularly relevant since Hg has been demonstrated to be toxic to ramie [38]. Figure 2 shows that the Ag levels in the ramie roots and leaves under SbIII and SbV stresses all significantly increased with the increasing addition of Ag. This indicated that Ag could be taken up by ramie and exert its inhibitory effect. 

Under SbIII stress, the addition of Ag remarkably reduced the Sb levels, both in the roots and leaves of ramie (Figure 2), suggesting that ramie might take up SbIII via aquaglyceroporins. This aligns with our previous transcriptomic analysis, which demonstrated a significant upregulation of the NIP1;2 gene in ramie roots under SbIII stress [13]. This upregulation suggests that NIP1;2 played a role in transporting SbIII. NIP1;2 belongs to the nodulin 26-like intrinsic protein (NIP) subfamily of the aquaglyceroporin family and is localized in the plasma membrane. Bienert et al. [39] also observed that *O. sativa* OsNIP2;1 was permeable for SbIII. Kamiya et al. [26] reported that NIP1;1 participated in SbIII transport in *Arabidopsis thaliana*. Our results are further supported by the findings of Feng et al. [19], who found that the addition of HgCl_2_ as an aquaporin inhibitor significantly reduced the Sb contents in rice roots and shoots. This suggested that the uptake of SbIII in rice might also be through aquaporins. Moreover, Tschan et al. [23] proposed that SbIII can passively cross the cell membrane via aquaglyceroporins and water.

The addition of Ag also resulted in a notable decrease in Sb levels in the roots exposed to SbV, but it did not cause any significant change in Sb levels in the SbV-exposed leaves (Figure 2). Although our previous transcriptomic results indicated that NIP1;1, NIP2;3, and NIP5;1 might participate in the transmembrane transport of SbV, no NIP transporters of SbV have been identified previously. Similarly, Feng et al. [19] also observed that the addition of HgCl_2_ significantly reduced the Sb concentration in the roots of rice treated with SbV. Tschan et al. [40] showed that the transport of SbV into the root symplast required anion transporters with low selectivity, enabling Sb(OH)^−^ to replace essential nutrient anions like Cl^−^ or NO_3_^−^. Therefore, it might also be the competition between Sb(OH)^−^ and NO_3_^−^ that leads to the decrease in Sb content. To determine whether the decrease in Sb concentration in ramie roots induced by Ag is a result of aquaglyceroporin inhibition or the influence of anions, further in-depth study on the functional characterization of aquaglyceroporins in ramie roots is needed. In addition, different from our results, Tisarum et al. [28] found that Ag addition did not affect the Sb concentrations in *Pteris vittata* gametophytes under SbIII and SbV stresses, suggesting that the aquaglyceroporin channel of *Pteris vittata* gametophytes was insensitive to Ag. A possible reason for this difference was that there were large differences between plant species that might lead to different uptake mechanisms for Sb.

### 3.4. SbV Analogs on SbV Uptake by Ramie

**Figure 3:**
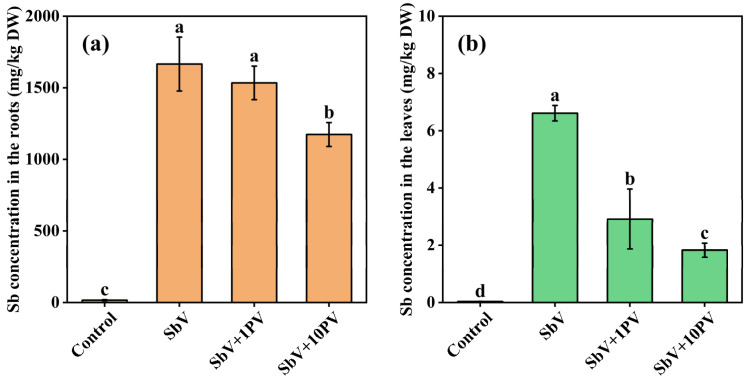
Effects of PV on Sb concentrations in the roots (**a**) and leaves (**b**) of ramie under SbV stress. Lowercase letters indicate statistically significant differences at *p* < 0.05. DW: Dry weight.

Considering the similarity in molecular sizes between SbV and PV [28], it is possible that they might compete for the same transporter during uptake, especially since they share similar characteristics as negatively charged oxyanions. The addition of 1 mg/L PV had no significant impact on Sb levels, while the addition of 10 mg/L PV significantly decreased Sb levels in the roots of ramie exposed to SbV (Figure 3). Both concentrations of PV addition significantly reduced the Sb concentration in SbV-treated ramie leaves. This was consistent with the results of Feng et al. [19], who also observed that the addition of PV at sufficient concentrations (5 and 10 mg/L) significantly decreased the Sb content both in rice roots and shoots under SbV stress. Tisarum et al. [28] demonstrated that the addition of PV had an inhibitory effect on SbV uptake when sufficient P was available. However, it did not influence SbV levels when Pteris vittata gametophytes had been subjected to P starvation for 12 weeks. Tschan et al. [21] observed that the addition of PV in the growth medium for *Helianthus annuus* L. and *Zea mays* L. did not lead to a reduction in the uptake of SbV, which is probably due to their lower PV addition concentration at 3 mg/L. Typically, P is absorbed by plant roots via both low-affinity and high-affinity P transporters [41]. High-affinity uptake systems dominate at lower substrate concentrations, whereas low-affinity uptake systems dominate at higher substrate concentrations. According to the speculation of Feng et al. [19], high levels of PV could compete with the uptake of SbV, indicating that the uptake of SbV might occur through a low-affinity P uptake system. Therefore, we also speculated that the uptake of SbV by ramie might be via low-affinity P transporters. However, considerable knowledge gaps persist regarding the mechanisms of SbV uptake and translocation. Additional research in the future will be necessary to validate the above speculations.

### 3.5. SbIII Analogs on SbIII Uptake by Ramie

**Figure 4:**
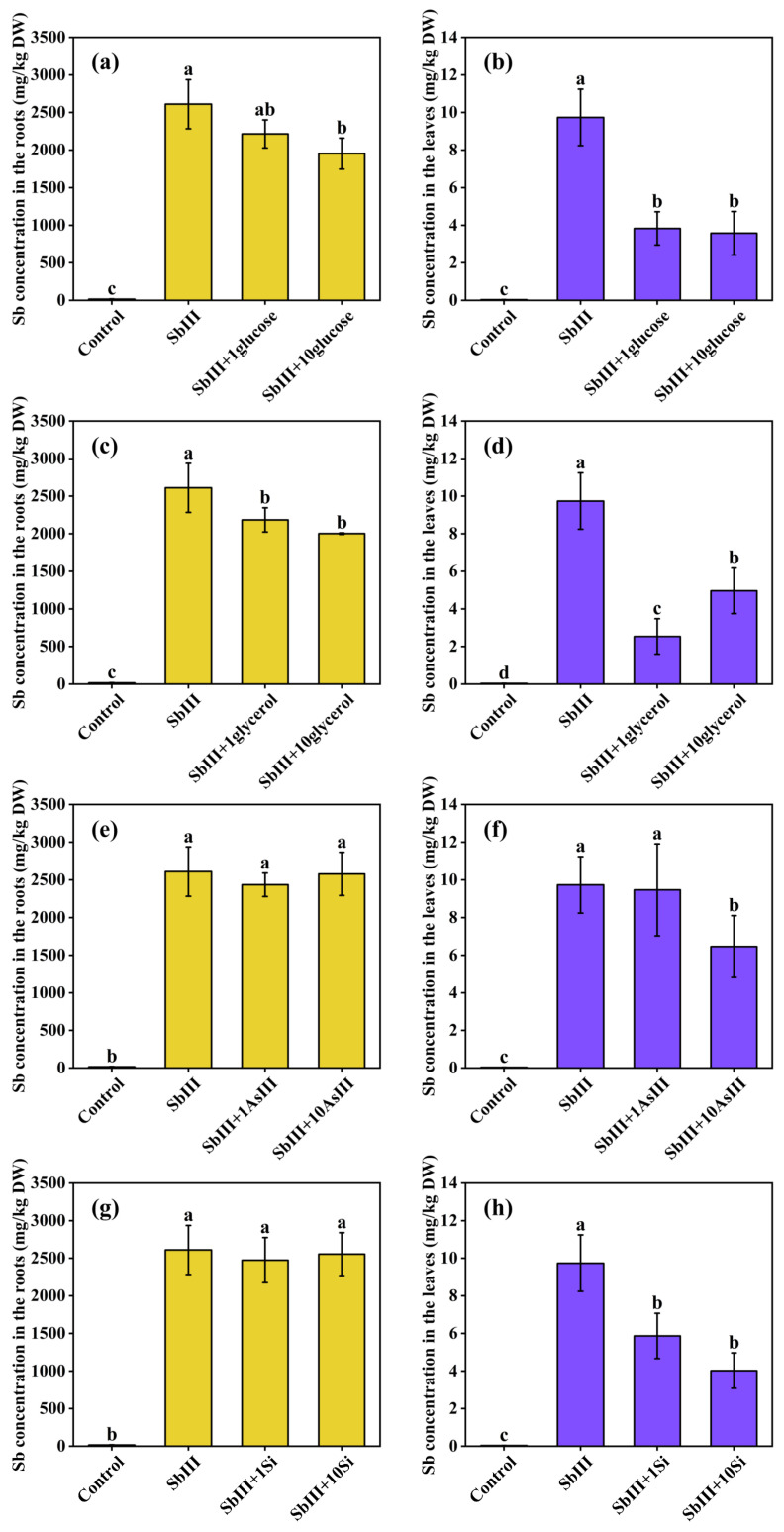
Effects of SbIII analogs on Sb concentrations in the roots (**a**,**c**,**e**,**g**) and leaves (**b**,**d**,**f**,**h**) of ramie under SbIII stress. Lowercase letters indicate statistically significant differences at *p* < 0.05. DW: Dry weight.

SbIII analogs, including AsIII, glycerol, glucose, and silicic acid, are oxyanions of comparable molecular size, and plants typically absorb these analogs through aquaglyceroporin channels, resulting in competition for uptake [28]. Figure 4 illustrates the effects of these four SbIII analogs on SbIII uptake by ramie. The addition of AsIII and glycerol both significantly reduced the Sb levels in ramie roots and leaves suggesting that SbIII and AsIII might compete for a similar uptake pathway in ramie. This might be because SbIII and AsIII exist as uncharged Sb(OH)_3_ and As(OH)_3_ in solutions at neutral pH ranges, which have similar structures to glycerol but with smaller molecular volumes [42]. These shared chemical properties are likely to be the basis for SbIII and AsIII being easily transported by the aquaglyceroporins, which are also permeable to glycerol [24,26]. Competition between SbIII and AsIII was also observed in rice (*Oryza sativa* L.) [25]. However, the addition of AsIII promoted the uptake of SbIII in As hyperaccumulators *Pteris vittata* L. [43], and SbIII uptake was not inhibited by AsIII and glycerol in *Pteris vittata* gametophytes [28]. This suggests that the uptake mechanisms of SbIII and AsIII are complex and vary depending on the plant species. Additionally, the Sb levels in ramie roots and leaves were also inhibited by silicic acid addition. Similarly, previous studies illustrated that the application of Si in rice reduced Sb accumulation and limited Sb translocation to shoots in rice (*Oryza sativa* L.) [44], giant reed (*Arundo donax* L.) [45], and maize (*Zea mays* L.) [46]. On the one hand, the Si-induced reduction in Sb contents in ramie tissues could be attributed to the competition between Sb and Si for the same uptake channels. On the other hand, it might be because Si could mitigate the toxicity of Sb and enhance the lignification of root structures, potentially restricting the translocation of Sb [45]. Different from AsIII, glycerol, and silicic acid, the addition of glucose had no significant effect on Sb concentrations in the roots and leaves. Similar results were also observed in *Pteris vittata* gametophytes [28]. Thus, ramie seemed to have different uptake pathways for glucose and SbIII.

## 4. Conclusions

In conclusion, ramie primarily transported Sb from its roots to the aboveground parts by increasing the concentration of Sb in the bleeding sap. Ramie exhibited a higher efficiency in SbIII uptake compared to SbV. The process of SbV uptake by ramie was energy dependent and relied on ATP for energy supply. Furthermore, SbV uptake by ramie might occur through low-affinity P transporters and compete with NO_3_^−^ for the uptake pathway. The inhibitory effects of Ag on SbIII uptake, along with the competitive interactions observed between SbIII and analogs such as AsIII, glycerol, and silicic acid, suggested that ramie likely utilized aquaglyceroporins for SbIII uptake. While these findings contribute to our understanding of Sb uptake pathways in ramie, it is crucial to acknowledge the need for further validation. In real contaminated soils, numerous interfering factors, including soil nutrients, physicochemical properties, pH, Eh, etc., can affect the uptake of Sb by ramie. Further pot experiments and field trials are necessary to validate the effectiveness of ramie in the phytoremediation of Sb-contaminated soils.

## Figures and Tables

**Table 1:** Treatment design.

Treatment	Inhibitor Concentration (mg/L)	Sb Concentration (mg/L)	
		SbIII	SbV
Control	0	0	0
SbIII	0	10	
SbV	0		10
SbIII + 1C_3_H_4_O_4_	1	10	/
SbIII + 5C_3_H_4_O_4_	5	10	/
SbIII + 10C_3_H_4_O_4_	10	10	/
SbV + 1C_3_H_4_O_4_	1	/	10
SbV + 5C_3_H_4_O_4_	5	/	10
SbV + 10C_3_H_4_O_4_	10	/	10
SbIII + 0.01Ag	0.01	10	/
SbIII + 0.1Ag	0.1	10	/
SbIII + 1Ag	1	10	/
SbV + 0.01Ag	0.01	/	10
SbV + 0.1Ag	0.1	/	10
SbV + 1Ag	1	/	10
SbV + 1PV	1	/	10
SbV + 10PV	10	/	10
SbIII + 1AsIII	1	10	/
SbIII + 10AsIII	10	10	/
SbIII + 1glycerol	1	10	/
SbIII + 10glycerol	10	10	/
SbIII + 1Si	1	10	/
SbIII + 10Si	10	10	/
SbIII + 1glucose	1	10	/
SbIII + 10glucose	10	10	/

**Table 2:** The weight of bleeding sap, the Sb concentration in the bleeding sap, and the absolute amount of Sb transported by ramie under SbIII or SbV stresses.

**Treatment**	**Bleeding Weight of Xylem Sap (g)**	**Sb Concentration in the Bleeding Sap (mg/kg)**	**Sb Transported (ng/h)**
Control	0.062 ± 0.010 a	4.127 ± 0.010 c	0.016 ± 0.002 c
SbIII	0.060 ± 0.015 a	123.0 ± 25.69 a	0.460 ± 0.104 a
SbV	0.066 ± 0.008 a	58.37± 11.50 b	0.242 ± 0.057 b

Note: Lowercase letters indicate statistically significant differences at *p* < 0.05. DW: Dry weight.

## Data Availability

Data sharing not applicable.
